# Tuberculous Meningitis in a Child: A Rare Presentation of Cytotoxic Lesion of the Corpus Callosum

**DOI:** 10.3390/tropicalmed10040096

**Published:** 2025-04-04

**Authors:** Ny Thi Hong Tran, Nhung Thi Hong Nguyen, Uyen Phuong Vo, Julie Huynh

**Affiliations:** 1 Oxford University Clinical Research Unit, Ho Chi Minh City 70000, Vietnam; jhuynh@oucru.org; 2 Neurology Department, Tam Anh General Hospital, Ho Chi Minh City 70000, Vietnam; 3 Paediatric Department, Pham Ngoc Thach Hospital, Ho Chi Minh City 70000, Vietnam; hongnhung200284@gmail.com (N.T.H.N.); phuonguyen19971997@gmail.com (U.P.V.); 4 Centre for Tropical Medicine and Global Health, Nuffield Department of Medicine, University of Oxford, Oxford OX1 2JD, UK

**Keywords:** tuberculosis, meningitis, splenium, corpus callosum

## Abstract

Tuberculous meningitis (TBM) is the most severe form of tuberculosis, disproportionately affecting vulnerable populations such as young children and people living with human immunodeficiency virus (HIV). Major challenges to accurate and early diagnosis of TBM are the non-specific clinical features which overlap with other infectious syndromes and the lack of adequately sensitive tests to detect *Mycobacterium tuberculosis* in the cerebrospinal fluid (CSF). Diagnosis is, therefore, still dependent on clinical suspicion along with clinical features, cerebrospinal fluid (CSF) characteristics and, where facilities are available, neuroimaging. Typical neuroimaging features of TBM include hydrocephalus, infarcts, tuberculomas and basal exudates; however, less well described are very rare features such as cytotoxic lesion of the corpus callosum (CLOCC), otherwise known as transient splenic lesion. We describe the first case report of a child with confirmed TBM who had a very rare presentation of CLOCC with complete recovery and present a literature review on the pathophysiology and alternative aetiologies where CLOCC is more commonly seen.

## 1. Introduction

An estimated 10.8 million people fell ill with tuberculosis (TB) in 2023, of which 1.25 million were children (aged 0–14 years) [1]. Children under five years of age have the lowest treatment coverage and suffer the highest mortality [2]. It is estimated that 24,000 children suffer from the most severe form of TB, TB meningitis (TBM), but only half are diagnosed and treated [3]. TBM is associated with substantial mortality and neurological sequelae and there is little evidence to guide optimal management to improve these outcomes. Diagnosis is dependent on clinical, cerebrospinal fluid (CSF) characteristics and where facilities are available, neuroimaging, as there is no single diagnostic test with adequate sensitivity [4,5].

TBM is a subacute/chronic meningitis which has insidious onset followed by focal neurological manifestations and eventual coma if not treated. Neurological signs occur in over half of children with TBM, which are most commonly an altered mental state and nuchal rigidity; however, these are late findings [6]. Cranial nerves II, III and VI are most commonly affected as they traverse the cranial floor where the characteristic basal exudates in TBM occur [7]. Brainstem signs are also not uncommon, with many displaying one or more signs of brainstem dysfunction [8,9]. Classically, CSF will have white cells in the hundreds, lymphocytic predominance, high protein (>1 g/L) and depressed CSF glucose (<2.2 mmol/L) or low CSF/blood glucose ratio (<0.5) [10,11,12]; however, only one third have all three CSF features on their first lumbar puncture [13]. A neutrophil predominance can occur in early disease and be mistaken for acute bacterial meningitis in about one third of those with TBM [11,13] and 10% have an absence of CSF pleocytosis [8]. Confirming a diagnosis of TBM requires detecting *M. tuberculosis* in CSF either by acid fast smear or rapid molecular tests such as PCR or mycobacterial culture, although the former has very low sensitivities outside of specialized research settings and the latter takes too long to impact clinical decision making [14]. Xpert or Ultra is recommended by the World Health Organization (WHO) as the initial test for extrapulmonary tuberculosis, including TBM; however, diagnostic sensitivities remain inadequate and fail to detect all cases [15]. A reliance on careful and thorough history taking, including TB contact history, searching for TB elsewhere and multiple investigative modalities, including neuroimaging, to diagnose TBM remain essential.

*Mycobacterium tuberculosis*, which enter via a haematogenous spread from the lungs, cause an inflammatory response in the meninges and brain. Inflammatory exudates at the basal cisterns and interperduncular region, in close proximity to cortical and meningeal blood vessels, proceed to cause a number of central nervous system (CNS) complications including vasculitis of small- and medium-sized blood vessels, which lead to infarcts and reduced reabsorption and obstruction of CSF flow, which lead to hydrocephalus [16]. The neuroimaging findings of basal exudates, hydrocephalus and infarct are not infrequently seen together as a classical triad, which increases the specificity for TBM [8,17,18]. A computed tomography (CT) brain scan is frequently accessible, and although it is the preferred modality in emergency settings, can be normal in up to 30% of TBM cases and is inferior to MRI for detecting infarcts, especially those involving the basal ganglia and brainstem, leptomeningeal nodules and small tuberculomas [19,20]. Our case report describes a presentation of a reversible splenic lesion, otherwise known as cytotoxic lesion of the corpus callosum (CLOCC), which is a very rare neuroimaging feature seen in TBM, usually seen in other pathologies and the first to be described in a child.

## 2. Case Report

**Figure 1:**
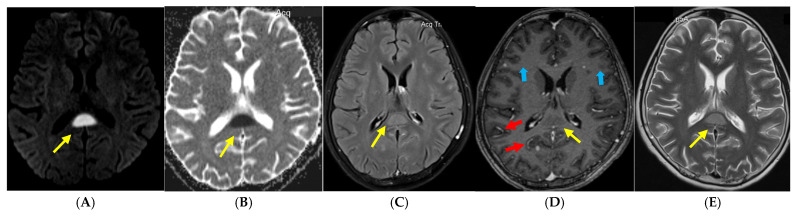
(**A**–**E**): Baseline MRI of a child with TB meningitis. Diffusion weighted (**A**), axial FLAIR (**C**) and Axial T2W (**E**) images showed a hyperintense, well-defined lesion in the splenium of the corpus callosum (yellow arrows). The same lesion was slightly hypointense on Axial T1W image and did not enhance with gadolinium (**D**). Corresponding lesion was hypointense on the apparent diffusion coefficient (ADC) map (**B**). Tuberculomas (blue arrows) and focal meningeal enhancement (red arrows) were also present.

**Figure 2:**
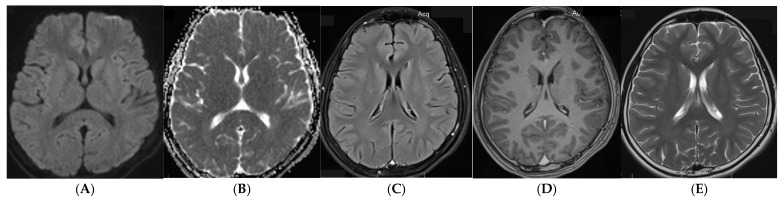
(**A**–**E**): Repeat MRI at 6 months of treatment. Diffusion weighted (**A**), ADC (**B**), axial FLAIR (**C**), Axial T1W post Gad (**D**) and Axial T2W (**E**) images show a complete absence of lesions previously seen in Figure 1.

A previously healthy 13-year-old male from rural Viet Nam was transferred to a tertiary hospital with a one-month history of persistent fever, poor appetite, weight loss, worsening headache and vomiting despite antipyretics and multiple courses of antibiotics in the community. There was no history of altered consciousness or seizures. He lived with his parents and younger sister who were all in good health and more recently was unable to attend school due to worsening headache. There was no known household exposure to tuberculosis (TB) nor contact with domestic or farm animals. Upon arrival, the child was of average weight (47 kg) but was lethargic. He was febrile (38.3 °C), had a heart rate of 104 bpm, respiratory rate of 20 and a blood pressure of 100/60. On presentation, he was cooperative and had a Glasgow Coma Score (GCS) of 15, equal and normal pupillary reactions and no neurological deficits. Cardiovascular, abdominal and lymph node examinations were unremarkable. CSF via lumbar puncture was clear and showed 295 white blood cells (41% Lymphocytes and 59% Neutrophils), increased protein (144 mg/dL) [normal < 40 mg/dL), low CSF glucose (1.19 mmol/L) [normal > 2.2 mmol/L] and a low CSF/blood glucose ratio (0.23) [normal ≥ 0.5]. Serum sodium was 129 mmol/L. Gram stain and bacterial cultures in blood and CSF were negative. Chest radiograph was normal. Baseline magnetic resonance imaging (MRI), taken on day one, revealed a well-defined hyperintense lesion in the splenium of corpus callosum (SCC), focal meningeal enhancement and multiple small tuberculomas without hydrocephalus, basal exudates or cerebral infarcts (Figure 1). The HIV antibody, Toxoplasma IgM/IgG and Cysticercus cellulosae antibody were all negative. CSF showed scant acid-fast bacilli on the Ziehl-Neelsen stain; Xpert MTB/RIF Ultra detected *M. tuberculosis* in low levels and rifampicin resistance was not detected. CSF cultures isolated *M. tuberculosis* after 18 days of incubation, which was fully susceptible to rifampicin, isoniazid, pyrazinamide, ethambutol and streptomycin on phenotypic drug-susceptibility testing. Smear, Xpert MTB/RIF Ultra and mycobacterial cultures were negative in expectorated sputum. He was commenced on a four drug anti-tuberculosis regimen, including corticosteroid therapy, and made a full recovery without complications. At the six month follow up, he was well without any evidence of neurological deficit; a repeat MRI at six months of treatment showed complete resolution of previous changes (Figure 2).

## 3. Discussion

Despite treatment, tuberculous meningitis (TBM) leads to death in 20% of children and long-term disability in half of survivors; without treatment, it is universally fatal [6]. The most important predictor of mortality and disability is early diagnosis; worse outcomes are associated with more advanced disease at presentation [6]. In the absence of an adequately sensitive test to detect *M. tuberculosis* in CSF, a combination of clinical features, CSF and neuroimaging findings, and confirmation of TB elsewhere remain essential for the diagnosis of TBM. The array of neuroimaging features in TB meningitis highlights the heterogeneity of disease. Whilst there are no pathognomonic features of TBM on neuroimaging, it can provide useful supplementary information, especially where there is clinical suspicion of TBM. The neuroinflammation, which occurs when *M. tuberculosis* enters the CNS, is responsible for the typical macroscopic features of basal meningeal enhancement, tuberculomas, infarcts and hydrocephalus, which can occur alone or in combination [21]. Tubercular abscesses are rare and limited to case reports only [22,23]. Tuberculous encephalopathy, also rare, occurs in young children who present with convulsion, a reduced conscious state and coma without nuchal rigidity or focal neurological deficit. Neuroimaging findings in these children show severe cerebral oedema and myelin loss of the white matter without the typical neuroimaging features of TBM [21]. There is an even more striking scarcity of literature on CLOCC in TBM and we describe here the first reported case in a child.

The term CLOCC has previously been known by a variety of terms, including mild encephalitis/ encephalopathy with reversible splenial lesion [24], reversible splenial lesion syndrome [25] or as transient splenial lesions [26]. It has now been proposed as CLOCC to better reflect the underlying pathophysiology and the concept that these lesions are not always strictly splenial, not always reversible and not always associated with encephalopathy [27]; however, nomenclature in the literature is inconsistent. Whilst most cases present with signs and symptoms of encephalopathy or encephalitis, there are cases where only a headache or fever without neurological signs or symptoms are present [28]. Our case did not have encephalopathy but did have a worsening headache with fever, had the classical pattern of a high T2 signal, a low T1 signal, restricted diffusion and a lack of contrast enhancement at the splenium of the corpus callosum [27] which was notably reversible as evidenced by a normal subsequent MRI. Whilst these neuroimaging findings are not typical of TBM, the concomitant meningeal enhancement, multiple small tuberculomas and, importantly, the presence of *M. Tuberculosis* in the CSF, confirms the diagnosis. Given the absence of known household exposure to TB and the age of our case, we propose that he acquired TB in a community setting where young adolescents with infectious TB often socialise and gather, e.g., schools [29].

The current consensus is that callosal lesions are caused by cytotoxic edema and result from a cascade of inflammatory cytokines and stimulated cells; however, the exact pathophysiology is not well understood [27]. It has been proposed that the initial insult causes macrophages to release cytokines (e.g., IL-1 and IL-6). This in turn leads to the recruitment of T-cells, break-down of the blood–brain barrier, production of TNF-α and activation of astrocytes [27]. The end result is a massive increase in excitatory neurotransmitter glutamate which causes a fluid shift into astrocytes and neurons, giving rise to cellular swelling and cytotoxic edema [27]. This can be due to trauma, inflammation, infection or metabolic derangement [30]. A possible reason for the preference of the splenium of the corpus callosum is the presence of a high density of oligodendrocytes expressing glutamate receptors and cytokine receptors [30]. In addition, postmortem pathological findings have shown intramyelinic edema, loss of fibrous astrocytes and microglial reactions, with minimal lymphocytic infiltration [31]. Most imaging lesions disappear without sequelae within three weeks, further supporting the hypothesis of cytotoxic edema [30].

The cytotoxic lesion of the corpus callosum is a clinico-radiological syndrome that typically manifests in children with pathologies other than tuberculosis. A recent systematic review summarising the aetiology and neuroimaging findings of CLOCC (324 references, 416 adult and 937 children) demonstrated that overarching pathological classes differed markedly between adults and children [32]. CLOCC in adults were commonly associated as drug/toxin-induced (26%), viral infections including SARS-CoV-2 and influenza (18%), cerebrovascular diseases (18%), bacterial infections (10%), including *Mycoplasma pneumoniae* and *Staphylococcus aureus*, and seizures (6%). Meanwhile, in children, callosal lesions were most frequently associated with viral infections, especially influenza and rotavirus (73%), bacterial infections, mostly mycoplasma (7%), while seizures (3%) and metabolic entities (3%) were less common. In a small proportion (4%) of children, no associated disease could be identified [32].

A few brief reports of CLOCC have been previously described in TBM; however, to date, all have been in adults with suspected or confirmed TBM [33,34]. One adult case had a short presentation of headache and fever without encephalopathy and had a focal splenial lesion, typical of CLOCC on day one of admission, which involuted from the periphery inwards on a follow up MRI scan on day 20 [26], whilst another showed radiological improvement by day 9 [33]. To date, CLOCC has not been reported in children with TBM. We describe here the first case report of TBM in a child with MRI features which were highly consistent with CLOCC. TBM is associated with dysregulated and heterogeneous host inflammation which, in part, contributes to the clinical outcome [35,36]. Cytokine inflammatory profiles have a role in TBM and differ in those with HIV co-infection [36,37]. Host genetics may also influence these inflammatory profiles [38] and contribute to various MRI phenotypes seen in TBM; however, this requires further investigation. It is therefore biologically plausible that CLOCC, as a result of cytokinopathy caused by *M. tuberculosis*, can occur in TBM as well. The incidence of CLOCC may be underestimated as cranial MRI is not readily accessible in low–middle income settings where TB burden is high, is more technically difficult in young children owing to the need for anaesthesia and therefore requires a skillset which is not always readily available in low-resourced settings or is performed too late in unwell patients when CLOCC would be expected to disappear. Owing to its convenience and accessibility, CT is often more commonly used in these settings; however, this, and the lack of neuroimaging at multiple time-points to demonstrate reversibility, is insufficient for the identification of CLOCC. Whilst we report the first case of CLOCC in a child with TBM, it is possible this lesion occurs more commonly than currently appreciated.

## 4. Conclusions

Classical neuroimaging features of TBM are well established and in cases of diagnostic uncertainty, can be a useful modality. Rare and atypical neuroimaging features, although not as well described, can occur, especially in settings where TB is common. CLOCC is a very rare manifestation of TBM which is more commonly seen in children with viral infections, and is an example of the wide heterogeneity of TBM disease.

## Data Availability

All available data relevant to this case have been included in the manuscript.
